# Predicted Brain Age in First-Episode Psychosis: Association with Inexpressivity

**DOI:** 10.3390/brainsci14060532

**Published:** 2024-05-24

**Authors:** Dean F. Salisbury, Brian M. Wulf, Dylan Seebold, Brian A. Coffman, Mark T. Curtis, Helmet T. Karim

**Affiliations:** 1 Department of Psychiatry, University of Pittsburgh, Pittsburgh, PA 15213, USA; 2 Department of Bioengineering, University of Pittsburgh, Pittsburgh, PA 15260, USA

**Keywords:** brain age, first-episode psychosis, cognitive function, schizophrenia

## Abstract

Accelerated brain aging is a possible mechanism of pathology in schizophrenia. Advances in MRI-based brain development algorithms allow for the calculation of predicted brain age (PBA) for individuals. Here, we assessed PBA in 70 first-episode schizophrenia-spectrum individuals (FESz) and 76 matched healthy neurotypical comparison individuals (HC) to determine if FESz showed advanced aging proximal to psychosis onset and whether PBA was associated with neurocognitive, social functioning, or symptom severity measures. PBA was calculated with BrainAgeR (v2.1) from T1-weighted MR scans. There were no differences in the PBAs between groups. After controlling for actual age, a “younger” PBA was associated with higher vocabulary scores among all individuals, while an “older” PBA was associated with more severe negative symptom “Inexpressivity” component scores among FESz. Female participants in both groups had an elevated PBA relative to male participants. These results suggest that a relatively younger brain age is associated with a better semantic memory performance. There is no evidence for accelerated aging in FESz with a late adolescent/early adult onset. Despite a normative PBA, FESz with a greater residual PBA showed impairments in a cluster of negative symptoms, which may indicate some underlying age-related pathology proximal to psychosis onset. Although a period of accelerated aging cannot be ruled out with disease course, it does not occur at the time of the first episode.

## 1. Introduction

Since the time of Kraepelin [1], psychotic disorders have been suggested to involve a progressive degenerative course, as reflected in his new term dementia praecox, or progressive dementia. Later, Bleuler [2] objected to the idea of an invariable dementing course, citing a number of cases that showed full or partial recovery, and renamed the syndrome schizophrenia (split mind). (See Kendler, Tabb, and Wright [3] for a history of the mind versus biology debate in the development of psychiatry as a medical specialty.) For most of the 20th century, there was little post-mortem evidence of neurodegeneration or brain volume reductions in schizophrenia, with the exception of well-documented increases in ventricular size, e.g., [4].

The dominant model became a static perinatal lesion (whether from the genetic mis-programming of cortical differentiation, insults in the third trimester such as maternal infection or famine, and birth complications, etc.), which laid the foundation for abnormalities in brain function during post-pubescent brain maturation, e.g., [5,6]. Still, several lines of evidence have suggested a possible progressive change in young adulthood in the disease. For example, Benes [7] showed that reduced interneurons and increased dopaminergic fibers interacted during late myelination to produce neurodevelopmental abnormalities in the prefrontal cortices. Building on the initial speculation of Feinberg [8], McGlashan and Hoffman [9] suggested that late neurodevelopmental synaptic pruning might play a role in schizophrenia-related pathology. Waddington, Scully, and Youseff [10] suggested that the early developmental origins of a disorder are not antithetical to active progressive disease during adulthood, pointing to the Alzheimer’s-like plaques and neurofibrillary tangles that develop at relatively young ages in Down’s syndrome, a genetic disorder. They suggested some brain areas were more susceptible to processes during normal aging in schizophrenia that appear as progressive lesions but reflect accelerated aging.

In the 1990s and early 2000s, increasing evidence suggested that there was a demonstrable decrease in gray matter (GM) around the time of the emergence of psychotic symptoms. These findings were made possible by advances in MR imaging, where 1 mm^3^ voxels (volume pixels) could now be recorded. Volumetric work in several laboratories demonstrated the presence of subtle progressive GM loss in individuals following the emergence of psychosis. For example, Gur et al. [11] showed progressive frontal cortex loss, Thompson et al. [12] showed progressive loss in the parietal, temporal, and frontal cortices, Lieberman et al. [13] showed progressive ventricle increases, Cahn et al. [14] showed the progressive loss of whole brain GM and increased ventricle volume, Kasai et al. [15,16] showed progressive left superior temporal gyrus loss following first psychotic break, Ho et al. [17] showed increased sulcal cerebral spinal fluid after first break, and Bachmann et al. [18] showed progressive frontal and temporal GM loss. In addition, several studies indicated that, among individuals at clinical high-risk for developing psychosis (CHR), individuals that transitioned to psychosis showed progressive cortical GM loss. For example, Pantelis et al. [19] showed progressive loss in the temporal and frontal cortices, and Job et al. [20] showed the largest progressive loss of the left temporal lobe in genetic high-risk individuals that transitioned to psychosis. All of these studies indicate progressive GM loss, likely greatest in the temporal and frontal cortices, respectively, around the emergence of psychosis, both before and after.

To date, there is no known mechanism for this progression. Several candidates have been proposed, such as a failure to stop adolescent synaptic pruning, e.g., [9], glutamate-induced excitotoxicity and dendritic regression short of apoptosis, e.g., [21], and oxidative stress, e.g., [22]. Referring back to Kraepelin’s idea of a progressive dementing disorder, a plethora of papers have posited that schizophrenia (the most severe psychotic disorder) is a consequence of accelerated aging, e.g., [23,24]. However, while some evidence is consistent with accelerated aging, it remains an unproven hypothesis with some conflicting findings (see review [25]).

A promising methodology for shedding light on this hypothesis of accelerated aging is comparing the “brain ages” derived from neurotypical individuals to those of individuals with psychosis. In a recent meta-analysis, Ballester et al. [26] suggested an increased brain age of around 3 years in schizophrenia sampled across illness durations. For example, Shahab et al. [27] reported a brain age gap (BAG) of ~8 years in a group of younger individuals with schizophrenia with about 11 years of illness duration (mean age ~33 years), but no gap in an older group of individuals with schizophrenia with about 40 years of illness duration (mean age ~64). A second sample with about 15 years of illness duration (mean age ~45) showed an approximately 6-year age gap. Likewise, Shnack et al. [28] suggested that individuals with psychosis were approximately 3 years older in terms of BAG than neurotypicals, with accelerated loss in the first few years that levelled off after approximately 5 years of illness. By contrast, a large meta-analysis [29] of 2803 individuals with schizophrenia versus 2598 neurotypical individuals against an independently developed brain age model indicated a ~3.5 years BAG in schizophrenia, although both groups were older than that predicted by the model. This meta-analysis found no effect of illness duration or age at onset (nor symptom severity), suggesting that this increased brain age occurred prior to the emergence of psychosis. On the other hand, Kaufmann et al. [30] found an increased brain age in schizophrenia (n = 1100) and schizophrenia-spectrum illnesses (n = 300), but not in CHR individuals (n = 94). It is of primary importance whether these BAGs are present at first psychosis, within 1 year from the onset of psychosis. As detailed above, significant GM loss occurs after psychosis onset, e.g., [12,15,16]. Hajek et al. [31] showed a greater brain age in first-episode schizophrenia-spectrum individuals, albeit younger (~2.5 years) than in the larger cross-sectional studies described above. Chung et al. [32] examined CHR individuals to test early versus late neurodevelopmental issues in those that transitioned to psychosis. They found that all CHR individuals (236 non-converters and 39 converters to psychosis) had a greater brain age than neurotypical individuals (n = 109). Among the converters, younger individuals had a significantly greater BAG (12–17), but older individuals (18–21) did not, and only the older individuals showed an increased trajectory of loss. These findings suggest both early and late neurodevelopmental deficits that are reflected in age of onset, with an early neurodevelopmental deficit in younger individuals and no accelerated aging in older individuals prior to psychosis onset. Of some importance, however, the meta-analysis of Ballester et al. [26] found a greater BAG with age, but also a greater brain age in first-episode psychosis. Thus, while the data support progressively accelerated aging after first psychosis, it is possible that accelerated aging exists before the emergence of psychosis.

In the current study, we examine the brain ages of 98 individuals within 6 months of their first clinical contact for a suspected psychotic disorder against 76 neurotypical individuals. If a significantly greater BAG was detected at first psychotic episode, then either neurodevelopmental abnormalities or the initiation of accelerated aging were likely to have occurred prior to the emergence of psychosis. By contrast, if a greater BAG was not detected in this large first-episode sample, then the acceleration of aging probably occurs post-psychosis onset. For consistency with the current literature, our first analysis determined brain age and BAG and compared them between groups. However, BAG derived from subtracting actual age from predicted brain age (PBA) has been criticized for being linearly dependent on actual age [33], such that any group differences in BAG may reflect differences in the distribution of age in the groups. Hence, our main comparisons comprised regression analyses on PBA, which are more statistically robust.

## 2. Materials and Methods

### 2.1. Participants and Study Design

We recruited participants as part of ongoing first-episode psychosis (FEP) studies in the Salisbury laboratory. The samples comprised 76 psychiatrically well participants as healthy controls (HC) and 91 individuals with FEP. The HCs were recruited through fliers and online advertisements in the local area. The HCs performed Axis I and Axis II screening and reported no first-degree relatives with a psychiatric disorder. The FEP individuals were recruited from the UPMC Western Psychiatric Hospital inpatient and outpatient services. Among the 91 FEP individuals, 70 received baseline diagnoses within the schizophrenia spectrum. This first-episode schizophrenia-spectrum (FESz) group comprised 47 individuals with diagnoses of schizophrenia (paranoid: n = 27; undifferentiated: n = 15; and residual: n = 5), 6 of schizoaffective disorder, 5 of schizophreniform disorder, and 12 of psychotic disorder Not Otherwise Specified. All FESz individuals had less than one year of lifetime antipsychotic medication exposure and less than one year since either their first clinical contact (emergency room visit or hospitalization) or first psychological or pharmacological treatment for psychosis (Table 1). No participant had neurological comorbidities, a history of concussion or head injury with sequelae, or a history of alcohol or drug addiction in the last 5 years. All participants performed a urine drug screen prior to scanning, although a history of alcohol and marijuana use was not a rule out. All participants underwent magnetic resonance imaging (MRI), cognitive testing, and an assessment of their demographic and clinical measures. All procedures were in accordance with the Declaration of Helsinki, and were approved by the University of Pittsburgh Institutional Review Board. All participants provided written informed consent and were paid for their participation. Data were collected under 3 protocols: Tim Trio MRI—PRO14010185, Cortical Cells, Circuits, Connectivity and Cognition in Psychosis, initial approval 3 March 2014; Prisma MRI—PRO16030020, Attentional Modulation of Sensory Signals in First Episode Psychosis, initial approval 24 June 2016; merged with STUDY19030077, Pathology and Pathophysiology in Early Psychosis, initial approval 28 June 2017.

The groups were matched for age, sex distribution, and premorbid IQ, estimated with the vocabulary subtest of the Wechsler Abbreviated Scale of Intelligence (WASI [34]). Diagnoses were based on the Structured Clinical Interview for DSM-IV interview [35] and chart review, and symptoms were rated using the Brief Psychiatric Rating Scale (BPRS [36]), the Scale for the Assessment of Negative Symptoms (SANS, [37]), and the Scale for the Assessment of Positive Symptoms (SAPS, [38]). Symptom clusters based on the SANS and SAPS items were calculated based on the hierarchical cluster analysis by Longenecker et al. [39]. The participants also performed the Matrix Reasoning test of the WASI and underwent MATRICS Consensus Cognitive Battery testing (MCCB [40]) to assess the current effects of their psychosis on their cognition. Social functioning was assessed with the Social Functioning Scale (SFS) and the Global Functioning: Social and Role scales (GF:S and GF:R [41]). All assessments were conducted by an expert diagnostician independent from the imaging laboratories.

### 2.2. MRI Acquisition

All participants underwent MR scanning at the MR Research Center of the University of Pittsburgh. Axial T1-weighted images were used to calculate PBA. In total, 61 individuals (36 FESz, 25 HC) underwent scanning on a 3T Siemens Tim Trio using a 32-channel phase array head coil. Sagittal T1-weighted anatomical MR images were obtained with a multi-echo 3D MPRAGE sequence (TR/multi-echo TE/TI = 2530/1.74, 3.6, 5.46, 7.32/1260 ms, flip angle = 7°, field of view (FOV) = 220 × 220 mm, 1 mm isotropic voxel size, 176 slices, GRAPPA acceleration factor = 2). A total of 85 individuals (34 FESZ and 51 HC) underwent scanning on a 3T Siemens MAGNETOM Prisma scanner using a 32-channel phase array head coil. Sagittal T1-weighted anatomical MR images were obtained with a 3D MPRAGE sequence [TR/TE/TI = 2400/2.22/1000 ms, flip angle = 7°, field of view (FOV) = 256 × 240 mm, 0.8 mm isotropic voxel size, 208 slices, GRAPPA acceleration factor = 2]. The participant distributions between scanners differed (chi-squared (df = 1) = 5.15, *p* = 0.023). Consequently, the scanner was controlled in subsequent analyses. The participants underwent approximately 1 h of scanning, but only the T1-weighted scans are presented here.

### 2.3. Data Availability

Data for individuals scanned on the Prisma are available on the US NIMH NDA database. All raw data are available upon request to the first author.

### 2.4. Brain Age Estimation

Brain age estimation was conducted using BrainAgeR (v2.1), an open-access algorithm for predicting brain age from raw T1-weighted scans (https://github.com/james-cole/brainageR, access dates: 1 May 2022 through 21 June 2022, [42]). Briefly, this algorithm conducted preprocessing and then prediction from a sample of 3377 healthy individuals (18–92 years old)—there was no additional training performed on the data from this study. In the preprocessing steps, structural scans were segmented and normalized to MNI space using SPM12 (https://www.fil.ion.ucl.ac.uk/spm/software/spm12, access dates: 1 May 2022 through 21 June 2022). Gray matter, white matter, and cerebrospinal fluid were first masked using a threshold of 0.3 from a mean image template generated from 200 scans (a subset of the 3377 individuals that included 20 individuals from 10 scanners). In the prediction steps, the algorithm was applied to the new data. This algorithm used principal components analysis to first retain 80% of the variance in brain volumes and estimated the ages using a Gaussian Process Regression. This resulted in 435 principal components that were then applied to our data to predict the ages. On independent data, this model has performed well.

### 2.5. Statistical Analysis

Brain age was predicted in R (v4.1.3). We first evaluated the performance of the brain age algorithm by evaluating the MAE and ensuring that this was approximately 5 years in the HCs. Other analyses were conducted in SPSS v28. Demographics, neuropsychological performances, and social functioning were compared using *t*-tests or chi-squared tests where appropriate. Correlations between age, PBA, and BAG are reported for each sample. Kolmogorov–Smirnov Z was used to test for similarity of age distributions between the samples.

Next, regression analyses between the PBAs (dependent variable) and the groups were conducted. Age, sex, scanner, and pSES were entered as control variables. Because pSES was not associated with PBA, subsequent regression models were adjusted for age, sex, and scanner. Subsequent regressions were conducted between PBA and the following sets of variables separately: (1) Premorbid IQ (WASI Vocabulary t-score); (2) MATRICS fluid cognitive measures sensitive to psychosis; (3) social functioning measures; and (4) for FESz only, symptom measures (SAPS and SANS component scores). Finally, for FESz, premorbid IQ and any significant symptom associates were included to ensure that symptoms but not IQ were associated with PBA. We examined Probability–Probability plots for normality and report the Variance Inflation Factor (VIF) for each model tested.

## 3. Results

### 3.1. Demographics, Neuropsychological, and Social Functioning

All means (s.d.), tests of significance, and effect sizes (Cohen’s d) are presented in Table 1. The groups did not differ in age, sex distribution, or WASI Vocabulary t-scores. FESZ had lower pSES than the HCs. Consistent with the effects of psychosis on cognition, the WASI Matrix Reasoning, MATRICS Composite Scores, and all MATRICS subtests were lower in FESZ. FESZ scored lower on all subscales of the SFS except Recreation. The current GF:S and GF:R, as well as the highest and lowest in the last year on both domains, were lower in FESZ. The clinical measures for FESz are presented in Table 2.

### 3.2. Predicted Brain Age and Brain Age Gap

**Figure 1:**
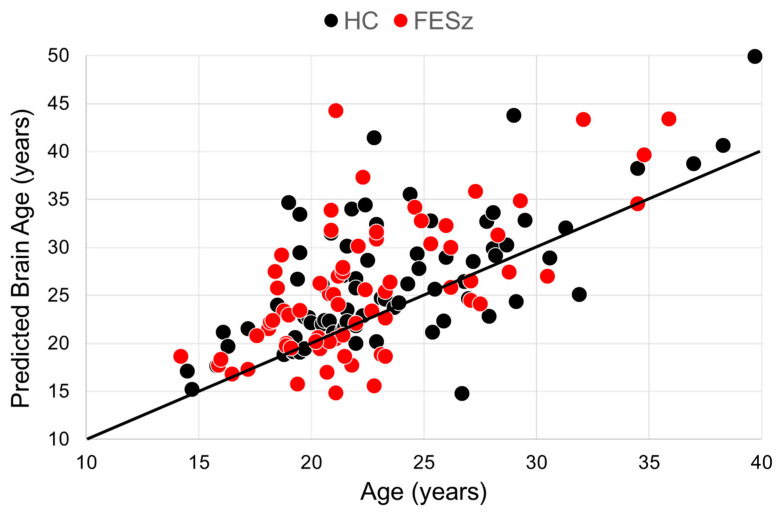
Correlations between predicted brain age and age. The solid line indicates perfect model prediction. Note the tendency for predicted ages to be older in both groups.

We found an MAE of 4.2 years in the HCs, indicating a good performance for the model and an MAE of 4.4 years in the whole sample. As mentioned above, age did not differ between the groups. The Kolmogorov–Smirnov test for distributions indicated that both groups were similarly distributed (Z = 0.974, *p* = 0.299). PBA and BAG did not differ significantly between the groups (Table 1). However, age correlated with PBA in both the HCs (r75 = 0.65, *p* < 0.001) and FESZ (r69 = 0.65, *p* < 0.001, Figure 1). In contrast, BAG did not correlate significantly with age in either the HCs (r75 = −0.131, *p* = 0.261) or FESZ (r90 = 0.006, *p* = 0.962).

#### 3.2.1. Regression Analyses

In our initial regression model, PBA was not associated with group (ß = −0.040, t = 0.616, *p* = 0.539, Table 3). For the following results, only significant associations are presented in the text. The full model results are presented in Table 4.

#### 3.2.2. Neuropsychological Performance

First, the WASI Vocabulary subtest estimates of premorbid IQ were tested. Among all participants, there was a significant association between premorbid IQ and PBA above and beyond the effects of age (ß = −0.163, t = 2.725, *p* = 0.007, Figure 1). In the model examining the MATRICS subtests (sensitive to the state effects of psychosis), there were no significant associations between PBA and neuropsychological tests. (See Table 4 for full results).

#### 3.2.3. Social Functioning

In the initial model with all social functioning measures, where no associations were detected, the GF:R Current and GF:R Lowest in the last year had VIFs over 30, and the GF:S Current and GF:S Lowest in the last year had VIFs over 16. They were removed from the model. In this second model, there were no significant associations between PBA and social functioning. (See Table 4 for full results).

#### 3.2.4. Symptoms

**Figure 2:**
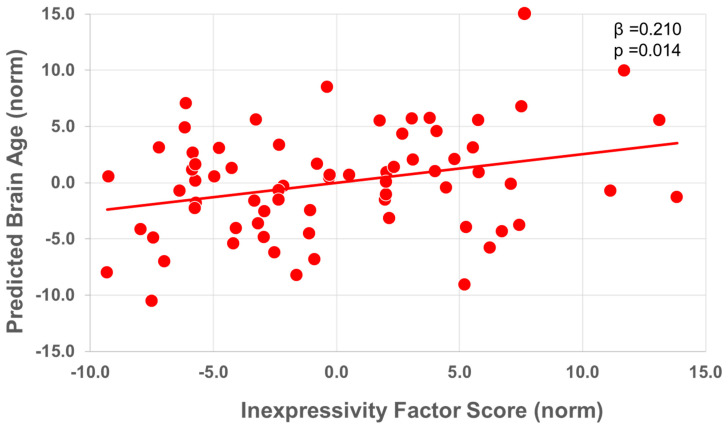
Relationship between predicted brain age and inexpressivity negative symptom component in first-episode schizophrenia-spectrum individuals. Partial regression plot of normalized values.

In FESZ only, the current symptom severity on the SANS/SAPS hierarchical clusters was regressed against PBA. In this model, above and beyond the effects of age, there was a significant association between an older PBA and poorer emotional inexpressivity (ß = 0.258, t = 2.472, *p* = 0.016, Figure 2). In a follow-up model in FESz, including the WASI Vocabulary subtest estimates of premorbid IQ, both IQ (ß = −0.178, t = 2.073, *p* = 0.042) and emotional inexpressivity (ß = 0.210, t = 2.532 *p* = 0.014) remained significantly associated with PBA. (See Table 4 for full results).

## 4. Discussion

Predicted brain age (PBA) and brain age gap (BAG) were examined in first-episode schizophrenia-spectrum individuals (FESz), as well as PBA’s associations with neuropsychological, social functioning, and symptom measures. FESz were tested relatively close to their first clinical contact for suspected psychosis, with a median of 27 days until scan. PBA and BAG were not elevated in FESz. Although the groups did not differ in age or age distribution, a more conservative regression model was used to assess for any PBA differences, adjusting for age, sex, and scanner. There was no association between PBA and group. These negative findings were associated with small effect sizes (Table 1), suggesting that the lack of difference was robust and not due to an underpowered study. In terms of accelerated aging prior to the onset of psychosis, these findings suggest little evidence for brain reduction in these individuals prior to the onset of psychosis. This is consistent with the findings of Chung et al. [32], where “older” CHR individuals (18–21) that converted to psychosis did not show an increased BAG, while younger individuals that converted to psychosis did. Chung et al. [32] also suggested an accelerated trajectory of gray matter loss in older individuals. Here, we did not see a correlation between BAG and age among FESz individuals, which would support a progressive course. This could be attributed to the relatively stable scan times among the FESz with respect to onset. However, the duration of untreated psychosis (DUP) did not correlate with PBA or BAG, nor did days from first contact to scan (all r values < 0.082). Thus, we can reasonably conclude that there was little evidence in this sample of 70 FESz compared to a matched group of 76 HCs for accelerated brain aging proximal to first clinical contact for psychosis. However, it is entirely possible that these FESz individuals were entering a post-psychosis period of accelerated aging. Only longitudinal follow-up testing of the cohort will provide definitive answers as to the individual trajectory of any accelerated brain aging in FESz.

Among all individuals, above and beyond the effects of age, a younger PBA was associated with higher estimates of premorbid IQ (WASI vocabulary performance). Vocabulary performance relies on verbal semantic memory, which is relatively robust against the effects of psychosis, unlike the domains assessed on the MATRICS, which are sensitive to the state effects of psychosis on cognition. The association between PBA and WASI Vocabulary performance was largely similar in FESz and HCs. Although perhaps not remarkable, possessing a younger brain age for one’s age has benefits for this cognitive domain among all individuals.

Within FESz, there were generally no associations between PBA and symptom severity above and beyond the effects of age. The exception to this was the negative symptom cluster of emotional inexpressivity. This cluster comprises SANS and SAPS items, including Relationships with Friends/Peers, Ability to Feel Intimacy/Closeness, Persecutory Delusions, Impersistence at Work/School, Recreational Interests and Activities, and Physical Anergia [39]. Although at first glance, these may seem disparate, they tend to vary together among individuals, suggesting sensitivity to pathology in an as of yet unknown common neural substrate. The results here suggest, once again beyond the effects of age itself, that an older PBA is associated with an increased severity of this negative symptom constellation. Importantly, including WASI Vocabulary scores in the final model indicated that the association with negative symptoms persisted and was not due to premorbid IQ.

Caveats include this being a relatively small sample of FESz, but this is <1% of the population once in their lives. Multi-center studies and mega-analyses of harmonized MRI data from individuals within 1 year of clinically significant psychotic symptoms would be beneficial. There are, at present, no longitudinal scans to examine PBA or BAG trajectory among these individuals. These data would be vitally important for determining whether late adolescent/early adulthood-onset FESz show an accelerated trajectory of aging. PBA models may suffer from regression to the mean errors (see [33]). Our groups were very well matched for age, and we performed alternative regressions to ameliorate such confounds. Samples were scanned on two different scanners. Adjusting for the scanner in the models may remove critical variance. We continue to collect participants on the newer scanner, but harmonization may be useful, albeit to the lowest common denominator. Parental socioeconomic status differed between the FESz and HCs. Although the models suggested there was no meaningful association, future samples should be matched to avoid potential confounds. The Variance Inflation Factor (VIF) was unacceptably high for the Current and Lowest in the last year Global Functioning scores. Although no associations were found, this suggests collinearity among the GF:S, GF:R, and SFS measures that may have affected the model sensitivity. However, the analysis of social functioning without these measures did not reveal any associations between social functioning and PBA. We chose to use a publicly available brain age model. It is possible that it was not optimal for our dataset, but we felt it important to use a validated model. Training a model on the HC group and testing group differences would be ‘double-dipping’, wherein we build a model on the controls and then test if there are differences between these groups on the model built on controls. The BrainAgeR model is a well-established and widely used model with a good performance, thus, we used this model to improve the generalizability of our results. In addition, while the MAE was less than 5 years, it is possible that a tighter-fitting model would be better. However, some previous evidence suggests that this may not be ideal, as this overfits pathological processes and prevents the detection of true clinical effects [43]. Future work developing a validated model with large sample sizes is necessary. Likewise, sex was associated with PBA, either significantly or at the trend level, in all regression models. Although females (n = 47, 22.8 ± 4.5 years) and males (n = 99, 23.2 ± 5.0 years) did not differ in age (t144 = 0.461, *p* = 0.645), the PBA was significantly greater in females than males (females: 27.8 ± 7.4 years, males: 25.3 ± 6.5 years, t144 = 2.047, *p* = 0.042). ANOVA with sex as a random factor and group as a fixed factor found no interaction between sex and group on PBA, so this is unlikely to reflect a differential sex effect in FESz. There is evidence that, even in healthy adults, this is a period of continued neural gray and white matter changes that differs between men and women [44]. The future development of brain age models that better account for sex would be beneficial. Among our HCs and FESz, we did not assess birth traumas, inflammatory markers, or specific genetic risk factors. These may be uncontrolled confounds. Future work with more detailed assessments of these measures is warranted. Finally, medications may affect cognitive performance, for better or for worse. However, we are ethically obligated to provide treatment, and a controlled wash-out period was not feasible.

In summary, PBA was associated in all individuals with a better semantic verbal memory, above and beyond the effects of actual age. There was no evidence for accelerated brain aging in these late adolescent/early adult-onset FESz. However, the negative symptom cluster of reduced emotional expressivity, along with several behavioral domains, was associated in FESz with a greater PBA, even when adjusted for actual age and premorbid IQ. Future work will examine these individuals during the early course of their psychotic illness to detect any increased trajectory of brain aging.

## Figures and Tables

**Table 1:** Demographic, neuropsychological functioning, and social functioning measures.

	FESz	HC			Cohen’s
	(N = 70)	(N = 76)	Statistic *	*p*-Value	d
Age	22.5 (4.5)	23.6 (5.1)	t_144_ = 1.37	0.172	0.227
PBA	25.6 (7.0)	26.5 (6.8)	t_144_ = 0.84	0.201	0.139
BAG	3.1 (5.3)	3.0 (5.2)	t_144_ = 0.16	0.877	0.026
Sex (F/M)	18/52	29/47	X^2^ = 2.59	0.108	
**pSES**	41.0 (13.8)	47.7 (11.6)	t_144_ = 3.19	**0.002**	**0.532**
					
WASI vocabulary	51.1 (9.7)	51.6 (6.5)	t_144_ = 0.34	0.738	0.055
**WASI matrix reasoning**	52.3 (8.7)	58.5 (5.7)	t_144_ = 4.58	**<0.001**	**0.759**
**MATRICS TMT**	40.0 (13.0)	48.4 (9.9)	t_142_ = 4.41	**<0.001**	**0.735**
**MATRICS processing speed**	39.2 (14.0)	52.4 (8.4)	t_142_ = 6.91	**<0.001**	**1.153**
**MATRICS attention**	36.4 (13.5)	47.8 (8.9)	t_142_ = 6.00	**<0.001**	**1.00**
**MATRICS working memory**	39.4 (13.5)	48.6 (8.7)	t_142_ = 4.94	**<0.001**	**0.825**
**MATRICS verbal learning**	43.6 (11.2)	52.7 (9.2)	t_142_ = 5.37	**<0.001**	**0.897**
**MATRICS visual learning**	39.7 (12.7)	46.1 (7.7)	t_142_ = 3.71	**<0.001**	**0.619**
**MATRICS reasoning**	44.2 (11.5)	51.2 (7.2)	t_142_ = 4.47	**<0.001**	**0.745**
**MATRICS social cognition**	41.6 (14.1)	54.5 (8.4)	t_142_ = 6.72	**<0.001**	**1.125**
**MATRICS composite**	34.9 (15.6)	50.6 (7.1)	t_142_ = 7.90	**<0.001**	**1.323**
					
**SFS Engagement/Withdrawal**	102.5 (11.8)	110.7 (10.4)	t_142_ = 4.42	**<0.001**	**0.737**
**SFS Interpersonal Interaction**	118.2 (18.5)	141.8 (8.0)	t_142_ = 10.03	**<0.001**	**1.674**
SFS Recreation	110.0 (13.4)	112.7 (9.7)	t_142_ = 1.38	0.170	0.231
**SFS Employment**	115.5 (7.7)	120.9 (4.3)	t_142_ = 5.21	**<0.001**	**0.870**
**SFS Independence Performance**	100.3 (11.5)	106.7 (9.0)	t_142_ = 3.74	**<0.001**	**0.624**
**SFS Independence Competence**	112.3 (11.8)	120.0 (7.1)	t_142_ = 4.82	**<0.001**	**0.803**
**SFS Prosocial**	108.4 (12.6)	112.1 (9.3)	t_142_ = 2.01	**0.046**	**0.335**
**GF:R current**	5.5 (2.2)	9.0 (0.1)	t_144_ = 13.50	**<0.001**	**2.237**
**GF:R lowest in last year**	5.4 (2.2)	8.9 (0.3)	t_144_ = 14.19	**<0.001**	**2.350**
**GF:R highest in last year**	7.4 (1.5)	9.0 (0.2)	t_144_ = 9.66	**<0.001**	**1.600**
**GF:S current**	5.4 (1.7)	9.0 (0.2)	t_144_ = 18.10	**<0.001**	**2.998**
**GF:S lowest in last year**	5.2 (1.8)	8.9 (0.3)	t_144_ = 18.22	**<0.001**	**3.017**
**GF:S highest in last year**	7.3 (1.2)	9.0 (0.1)	t_144_ = 12.10	**<0.001**	**2.004**

Note: Significant group differences are **bolded**. * differences in d.f. indicate missing data. For pSES, 2 individuals were in foster/state care, and no value was entered. PBA = Predicted Brain Age. BAG = Brain Age Gap. pSES = parental SocioEconomic Status. TMT = Trail Making Test.

**Table 2:** Clinical Measures in FESZ participants.

Test	Score
BPRS Total score	47.20 (9.53)
	
SANS/SAPS-derived components	
Auditory Hallucinations	3.37 (3.21)
Unusual Perceptions	0.93 (1.33)
Delusions	5.95 (3.87)
Thought Disorder	0.23 (0.34)
Inattention	2.17 (2.10)
Inexpressivity	7.43 (5.76)
Apathy/Asociality	7.57 (3.94)
	
Medicated/Unmedicated	54/16
Medication dosage (CPZ equivalents)	222.40 (122.17)
Mean days from first clinical contact to scan	53.26 (71.2)
Median days from first contact to scan	27
	
Mean months DUP	23.17 (38.53)
Median months DUP	6.89

**Table 3:** Predicted brain age (PBA) regression with group.

Variable	B	ß	t-Statistic	*p*-Value	VIF
**Age**	0.886	0.624	9.703	**<** **0.001**	1.080
**Scanner**	1.902	0.136	2.111	**0.037**	1.096
**Sex**	−2.865	−0.196	3.126	**0.002**	1.031
pSES	−0.016	−0.031	0.478	0.633	1.085
Group (FESz vs. HC)	−0.278	−0.04	0.616	0.539	1.133

Note: The B represents unstandardized coefficients, while ß is a standardized coefficient. Significant effects are bolded. VIF = Variance Inflation Factor. pSES = parental socio-economic status. FESz = first-episode schizophrenia-spectrum individuals. HC = healthy comparison individuals.

**Table 4:** Full model results of predicted brain age (PBA) associations with neuropsychological, social functioning, and clinical measures.

Variable	B	ß	t-Statistic	*p*-Value	VIF
**Premorbid IQ model**					
**Age**	0.887	0.623	10.093	**<** **0.001**	1.072
Scanner	1.573	0.113	1.833	0.069	1.074
**Sex**	−2.692	−0.184	3.074	**0.003**	1.004
**WASI vocabulary t-score**	−0.138	−1.630	−2.725	**0.007**	1.005
					
**MATRICS battery model**					
**Age**	0.969	0.684	10.381	**<** **0.001**	1.207
Scanner	1.678	0.121	1.911	0.058	1.121
**Sex**	−2.626	−0.180	−2.891	**0.004**	1.079
Trail Making Test	−0.076	−0.136	−1.231	0.221	3.392
Speed of Processing	0.087	0.167	1.228	0.221	5.144
Attention and Vigilance	−0.036	−0.065	−0.761	0.448	2.011
Working Memory	−0.032	−0.055	−0.564	0.574	2.697
Verbal Learning	0.047	0.077	0.940	0.349	1.850
Visual Learning	−0.058	−0.092	−1.146	0.254	1.810
Reasoning and Problem Solving	−0.108	−0.157	−1.923	0.057	1.859
Social Cognition	0.013	0.025	0.366	0.715	1.282
					
**Social Functioning Model**					
**Age**	0.829	0.584	7.973	**<** **0.001**	1.379
**Scanner**	2.180	0.158	2.381	**0.019**	1.137
Sex	−1.972	−0.136	−1.908	0.059	1.296
SFS Social Engagement/Withdrawal	0.056	0.097	1.307	0.193	1.411
SFS Interpersonal Interaction	−0.062	−0.169	−1.871	0.064	2.093
SFS Recreation	−0.047	−0.080	−1.100	0.328	1.403
SFS Employment Occupation	0.054	0.053	0.698	0.486	1.508
SFS Independence Performance	0.068	0.108	1.282	0.202	1.825
SFS Independence Competence	0.007	0.011	0.129	0.898	1.752
SFS Prosocial	0.029	0.048	0.640	524	1.543
GF:R Highest in Last Year	−0.416	−0.080	−0.674	0.502	3.657
GF:S Highest in Last Year	0.448	0.082	0.662	0.509	3.936
					
**Symptom Models (FESz only)**					
**Age**	1.041	0.669	7.582	**<** **0.001**	1.117
Scanner	0.729	0.053	0.588	0.559	1.155
**Sex**	−4.763	−0.301	−3.353	**0.001**	1.159
Auditory Hallucinations	0.019	0.009	0.088	0.930	1.378
Unusual Perceptions and Behaviors	−0.773	−0.147	−1.556	0.125	1.285
Delusions	0.262	0.145	1.355	0.181	1.653
Thought Disorder	0.863	0.043	0.438	0.663	1.355
Inattention	0.343	0.103	0.997	0.323	1.545
**Inexpressivity**	0.312	0.258	2.472	**0.016**	1.568
Apathy and Asociality	−0.150	−0.085	−0.857	0.395	1.406
					
**Age**	1.038	0.667	8.006	**<0.001**	1.091
Scanner	0.900	0.065	0.769	0.444	1.091
**Sex**	−4.035	−0.255	−3.053	**0.003**	1.066
**WASI Vocabulary t-score**	−0.128	−0.178	−2.073	**0.042**	1.121
**Inexpressivity**	0.254	0.210	2.532	**0.014**	1.054

Note: The B represents unstandardized coefficients, while ß is a standardized coefficient. Significant effects are in bolded. VIF = Variance Inflation Factor. WASI = Wechsler Abbreviated Scale of Intelligence. MATRICS = Measurement and Treatment Research to Improve Cognition in Schizophrenia (MATRICS) cognitive battery. SFS = Social Functioning Scale. GF:R = Global Functioning: Role. GF:S = Global Functioning: Social. FESz = first-episode schizophrenia-spectrum individuals. Emotional Inexpressivity is derived from hierarchical principal component analysis in Longenecker et al. (2022) [39]. WASI = Wechsler Abbreviated Scale of Intelligence.

## Data Availability

Portions of the data are available in the US NIMH NDA database. The original data was obtained elsewhere, are available from the first author upon appropriate request.
